# A Six-Gene Prognostic and Predictive Radiotherapy-Based Signature for Early and Locally Advanced Stages in Non-Small-Cell Lung Cancer

**DOI:** 10.3390/cancers14092054

**Published:** 2022-04-19

**Authors:** Javier Peinado-Serrano, Álvaro Quintanal-Villalonga, Sandra Muñoz-Galvan, Eva M. Verdugo-Sivianes, Juan C. Mateos, María J. Ortiz-Gordillo, Amancio Carnero

**Affiliations:** 1 Instituto de Biomedicina de Sevilla, IBIS, Hospital Universitario Virgen del Rocío, Consejo Superior de Investigaciones Científicas, Universidad de Sevilla, Avda. Manuel Siurot s/n, 41013 Seville, Spain; jvrr18@gmail.com (J.P.-S.); smunoz-ibis@us.es (S.M.-G.); everdugo-ibis@us.es (E.M.V.-S.); 2 CIBERONC, Instituto de Salud Carlos III, 28029 Madrid, Spain; 3 Department of Radiation Oncology, Hospital Universitario Virgen del Rocío, Avda. Manuel Siurot s/n, 41013 Seville, Spain; mjortizgordillo@yahoo.es; 4 Molecular Pharmacology Program, Memorial Sloan Kettering Cancer Center, New York, NY 10065, USA; quintaa1@mskcc.org; 5 Radiation Physics Department, Hospital Universitario Virgen del Rocío, Avda. Manuel Siurot s/n, 41013 Seville, Spain; jcmateos@us.es; 6 Departamento de Fisiología Médica y Biofisica, Universidad de Sevilla, 41013 Seville, Spain

**Keywords:** NSCLC, biomarkers, radiation oncology, prognosis, predictive signature

## Abstract

**Simple Summary:**

The search for prognostic and/or predictive gene signatures of the response to radiotherapy treatment can significantly aid clinical decision making. These signatures can condition the fractionation, the total dose to be administered, and/or the combination of systemic treatments and radiation. The ultimate goal is to achieve better clinical results, as well as to minimize the adverse effects associated with current cancer therapies. To this end, we analyzed the intrinsic radiosensitivity of 15 NSCLC lines and found the differences in gene expression levels between radiosensitive and radioresistant lines, resulting in a potentially applicable six-gene signature in NSCLC patients. The six-gene signature had the ability to predict overall survival and progression-free survival (PFS), which could translate into a prediction of the response to the cancer treatment received.

**Abstract:**

Non-small-cell lung cancer (NSCLC) is the leading cause of cancer death worldwide, generating an enormous economic and social impact that has not stopped growing in recent years. Cancer treatment for this neoplasm usually includes surgery, chemotherapy, molecular targeted treatments, and ionizing radiation. The prognosis in terms of overall survival (OS) and the disparate therapeutic responses among patients can be explained, to a great extent, by the existence of widely heterogeneous molecular profiles. The main objective of this study was to identify prognostic and predictive gene signatures of response to cancer treatment involving radiotherapy, which could help in making therapeutic decisions in patients with NSCLC. To achieve this, we took as a reference the differential gene expression pattern among commercial cell lines, differentiated by their response profile to ionizing radiation (radiosensitive versus radioresistant lines), and extrapolated these results to a cohort of 107 patients with NSCLC who had received radiotherapy (among other therapies). We obtained a six-gene signature (*APOBEC3B*, *GOLM1*, *FAM117A*, *KCNQ1OT1*, *PCDHB2*, and *USP43*) with the ability to predict overall survival and progression-free survival (PFS), which could translate into a prediction of the response to the cancer treatment received. Patients who had an unfavorable prognostic signature had a median OS of 24.13 months versus 71.47 months for those with a favorable signature, and the median PFS was 12.65 months versus 47.11 months, respectively. We also carried out a univariate analysis of multiple clinical and pathological variables and a bivariate analysis by Cox regression without any factors that substantially modified the HR value of the proposed gene signature.

## 1. Introduction

Lung cancer greatly influences the lives of patients and healthcare systems since it has the largest incidence and a nearly 95% mortality worldwide [1]. Based on its histological characteristics, it is divided into two large groups: small cell lung cancer (small-cell) and non-small-cell lung cancer (NSCLC). The latter accounts for approximately 85–90% of the total. In turn, this group is subdivided, according to histological and molecular characteristics, into the following: adenocarcinomas (the majority), squamous, large cells, neuroendocrine, and not otherwise specified. Currently, three clinical scenarios are considered regarding cancer treatment in newly diagnosed patients: resectable, locally advanced (not resectable), and metastatic. As shown in the main international therapeutic guidelines [2], radiotherapy treatment plays an important role in all settings. It is an alternative to surgical treatment in early stages (Stages I and II without nodal load) using stereotactic body radiotherapy (SBRT) and is complementary after surgical resections with affected edges and/or a positive nodal load (N2). In locally advanced stages (Stage III), where surgical resection is not possible, normofractionated radiotherapy treatment together with chemotherapy (concomitant or sequential) are the therapeutic standard. In patients with 1–5 thoracic and/or extrathoracic lesions, the option of local treatment with radical intention is considered an effective alternative. Last, most patients with metastatic spread in the central nervous system receive protocolized radiotherapy treatment if their health allows it. Focusing on unresectable locally advanced stages (Stages IIIA–C), there is currently no standard radiochemotherapy regimen, although the combination of a platinum-based regimen and chest radiotherapy has significantly improved the survival of these patients. These patients are treated with standard radiation doses of 60–66 Gy concomitantly, or sequentially, with combined chemotherapy [3,4,5]. Fractions can vary, but generally 1.8–2 Gy/fraction/day is used (normofraction). Despite the application of a combined cytotoxic treatment, we continue to observe local relapse rates of 30–50% in this group of patients [6,7]. This fact justifies, by itself, the need to continue delving into the biological keys that govern the poor clinical results obtained to date.

Research focused on improving antineoplastic therapies in lung cancer has been based on the genomic and proteomic study of tumors with a specific known genetic basis, such as EGFR and KRAS mutations. This molecular classification influences the response to biological therapies based on monoclonal antibodies and tyrosine kinase inhibitors in patients with lung cancer [8,9,10]. On the other hand, a nonuniform response of patients to these therapies has been observed, which suggests a resistance model that could be mediated by other mutations in some relevant genes (insertions in EGFR [11] or KRAS [12], amplification of MET [13,14] or mutations in the HER-242 kinase domain). We also know that the tumor genetic profile has a relevant impact on the response to chemotherapy [15] and radiotherapy [16,17]. There are currently several studies that relate some of the mentioned mutations to the mechanisms of radioresistance or radiosensitivity in NSCLC [17,18].

However, today, there are still no biomarkers to consider as a condition for radiotherapy treatment to be administered, despite knowledge of the effects that some mutations could have on the response to ionizing radiation. Thus, the classification of NSCLC based exclusively on the clinicopathological characteristics was the only determinant of the therapy administered. The availability of biological material and advances in transcriptomic analysis techniques have made it possible to improve the subclassification of the group of neoplasms encompassed within NSCLC [19,20,21,22].

Based mainly on a transcriptomic analysis, many studies have proposed different gene signatures in adenocarcinoma [23,24,25,26,27,28,29,30,31], squamous cell carcinoma [32,33,34], or NSCLC in general [35,36,37,38,39,40,41,42,43,44,45,46]. Some of these studies have attempted to identify the prognostic and predictive biomarkers of the response to systemic treatments. Most focused on the identification of markers that help in clinical decision making, regarding the suitability of administering adjuvant systemic treatment in the early stages of NSCLC after surgery [47,48]. In contrast, very few published studies suggest predictive signatures of response to ionizing radiation in NSCLC. Thus, based on the research work carried out by the American National Cancer Institute (NCI)—in vitro analyses of the biological effects of different antineoplastic drugs in cell lines [49,50,51,52,53,54,55]—Scott et al. published, in 2017, a study proposing a model to adapt the radiotherapy prescription to the individual sensitivity of each patient’s tumor. The model, called GARD (genome-based model for adjusting radiotherapy dose), combines information derived from the radiosensitivity index (RSI) and the linear quadratic model (LQ model) [56]. On the other hand, some of the miRNAs identified to date have been considered diagnostic and prognostic biomarkers in numerous neoplastic entities, including NSCLC [57,58]. Sun et al. [59] proposed a total of 11 miRNAs, which, together with variables such as stage, age, radiation dose administered, systemic treatment, and Karnofsky general state scale, were used to determine the DFS of each patient, but it was not possible to demonstrate its predictive capacity on the local control of the disease in a statistically significant way [59]. Finally, there are other publications where the generation of prognostic signatures is considered through the joint consideration of multiple nonspecific biomarkers of lung cancer and multiple clinical variables [60].

Given the limited scientific evidence published, we consider that the search for prognostic and/or predictive gene signatures of the response to radiotherapy treatment can significantly help with clinical decision making. These signatures can condition the fractionation, the total dose to be administered, and/or the combination of systemic treatments and radiation. The ultimate goal is to achieve better clinical results, as well as to minimize the adverse effects associated with current cancer therapies. To this end, we carried out a basic translational study, which, by analyzing the intrinsic radiosensitivity of 15 NSCLC lines, allowed us to obtain the differences in gene expression levels between radiosensitive and radioresistant lines, resulting in potentially applicable gene signatures in NSCLC patients.

## 2. Materials and Methods

### 2.1. Cell Lines

Fifteen NSCLC tumor cell lines from the American Type Culture Collection (ATCC) were used. Three corresponded to SCC (H226, H520, and Calu 1), two to bronchioloalveolar carcinomas (H1437 and H358), one to large cell carcinoma (H460), and nine were adenocarcinomas (A549, Calu-3, H1650, H1781, H1975, H2009, H2228, H3122, and HCC827). A RPMI culture medium with FBS and antibiotic was used, except for A549 (DMEM+sodium pyruvate, HEPES, and nonessential amino acids), Calu 1 (McCay + glutamine supplement), and Calu 3 (DMEM), according to the ATCC’s recommendations. Table S1 summarizes the histological information and molecular profile of some relevant NSCLC mutations of the cell lines used.

### 2.2. Clonogenicity Test for the Response to Ionizing Radiation by Determining the SF2 Parameter

We seeded the cells in six-well plates and allowed them to grow until they reached 70–90% confluence. Then, we irradiated the cells in a linear photon accelerator of 6 Mv of energy at a rate of 400 monitor units (MU) per minute. After changing the medium and incubating for 36–48 h, we counted the cells and seeded them in 10-cm-diameter culture plates, between 300 and 2000 cells per plate, in triplicate for each dose of radiation used (0, 2, 4, 6, and 8 Gy). We incubated the cells for a period of 7–25 days in a standard atmosphere to allow the formation of colonies. We scanned the plates and counted the number of CFUs using the ImageJ^®^ (NIH, Bethesda, MD, USA) computer program. The number of CFUs was normalized to the number of cells seeded in each case. Calculation of the radiobiological parameters was conducted as follows: (a) plating efficiency (PE): PE = number of colonies counted/number of cells seeded; and (b) surviving fraction at 2 Gy (SF2): SF2 = (number of colonies counted/number of cells seeded)/PE 9. The preparation of survival curves was based on the values of the surviving fraction in the control plates and those treated with 2, 4, 6, and 8 Gy.

### 2.3. RNA Extraction from Cells

A commercial miRNA Extraction Kit (miRNeasy^®^ Mini Kit (Qiagen, Barcelona, Spain)) was used following the manufacturer’s instructions.

### 2.4. cDNA Microarrays

The RNA obtained was subjected to a reverse-transcription reaction in the presence of six nucleotide random primers (Invitrogen/Life Technologies, Carlsbad, CA, USA) using Cy3- or Cy5-labeled dCTPs (GE Healthcare, Chicago, IL, USA) and MultiScribeTM RT (Thermo Fisher Scientific, Waltham, MA, USA). RNA from normal lung epithelial cells was used as a reference. Equal amounts of cDNA were used to make the microarray. Prehybridization was performed in a humidified chamber at 42 °C for 16–20 h, and the hybridization was performed at 65 °C in a GeneTac Hybridization Station (Genomics Solutions, Oberhaching, Germany). Hybridized samples were analyzed with a confocal scanner (ArrayExpress, PerkinElmer, Waltham, MA, USA), and the data were quantified using QuantArray software (PerkinElmer, Waltham, MA, USA). The level of significance of the expression of each gene was determined using a Student’s *t*-test (10,000 permutations), using, as a correction, the false discovery rate (FDR) test to eliminate false positives.

### 2.5. Transcriptome Analysis

After image acquisition and quantification, the mean signal intensity between replicates was determined for each sequence. At QT-02 (replicates 3–6 times represent the same gene), values with low signal intensity and low reproducibility between repeats were excluded (mean ± 2 standard deviations cutoff). The quantized signals were subjected to logarithmic and standardized transformation. We used a Student’s *t*-test of permutation (10,000 permutations) to determine the level of significance of the expression of each individual gene, and the false-positive rate was used as a correction for multiple analyses. Hierarchical cluster analysis and clustering reliability were evaluated using bootstrap techniques employing TMEV software (NIH, Bethesda, MD, USA). We identified altered mRNAs in cells that were resistant versus nonresistant to radiotherapy using targeted clusters, with subsequent enrichment of pathways. This process was carried out with the help of the bioinformatics service of the Institute of Biomedicine of Seville (IBIS).

### 2.6. Patient Cohort

The publicly available Cancer Genome Atlas (TCGA) cohort focused on patients with NSCLC (version 2018) was used. Stage I to III patients were selected from this cohort based on TNM classification (tumor, lymph nodes, metastasis) at the time of diagnosis. The main requirement for the selection was to have received radiotherapy treatment. For the analysis of the impact of clinical/pathological variables on OS and PFS, univariate analysis by Cox regression was performed. Subsequently, we carried out a bivariate analysis with the variable “gene signature” as the main element and considered the rest of the variables as independent to identify any modifying relationship or confounding variables, which would condition the impact of the gene signature on the OS or SLP. We used the statistical package SPSS^®^ version 20 (IBM, New York, NY, USA). To obtain the gene signatures, RNA sequencing data of tumor samples from TCGA patients were used, whose expression values were obtained as transcripts per million (TPM) and were subsequently transformed on a logarithmic scale.

### 2.7. Statistical Packages and Analysis

Survival analysis was performed using R software version 3.6.3 (San Francisco, CA, USA, 29 February 2020) with the Survival package (version 3.2.7) for Cox regression, Survminer (version 0.4.6) for Kaplan–Meier curves, Glmnet (version 3.0.2) for cross-validation and obtaining signatures and SurvivalROC (version 1.0.3) for ROC curves.

After identifying the differentially expressed genes between radiosensitive and radioresistant cell lines, we made use of RNA-seq data from the selected TCGA cohort. Next, a univariate Cox regression survival analysis was carried out. After selecting those genes whose impact on survival was statistically significant (*p* < 0.05), a Kaplan–Meier analysis of survival estimation was carried out, establishing the cutoff value of the median expression of each gene. We assessed differences in OS/PFS with a log-rank test. We selected those genes that were statistically significant in both analyses (*p* < 0.05) as potentially relevant elements for OS or PFS.

### 2.8. Obtaining Gene Signatures

**Figure 1:**
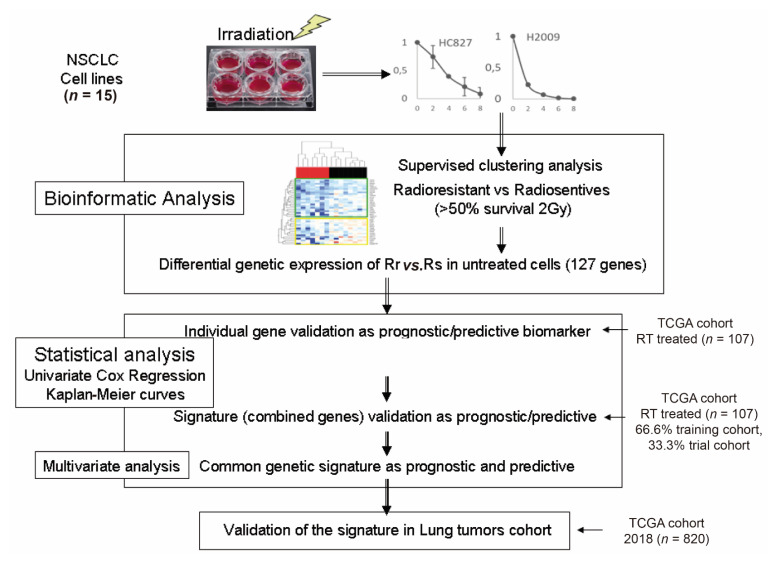
Diagram of the steps followed in the present work.

From the genes selected as individual elements with prognostic and/or predictive impact (HR with *p* < 0.05 and log-rank test with *p* < 0.05), we proceeded with a multivariate Cox regression analysis with L1 or Lasso type regularization. To do this, we separated the cohort of 107 TCGA patients into (1) a training cohort and (2) a trial cohort. We next carried out a cross-validation analysis of three iterations, randomly separating, for a maximum of 1000 times, 66.6% of the cohort for the training cohort and the remaining 33.3% for the test set. The prognostic and predictive signatures were constructed based on the linear combination of the regression coefficient obtained from the coefficients derived from the Cox regression analysis with L1 (β)-type regularization multiplied by the expression level of each gene (in units of transcripts per million). In this case, both the training and trial cohorts came from the same selected TCGA cohort. To calculate the survival prediction, we performed ROC curve (at 12, 24, and 60 months) and Kaplan–Meier analyses. Finally, we performed a Mann–Whitney U test to determine whether the differences in the expression of the genes of each signature in each risk group were statistically significant. Next, we separated the cohort based on histological type (adenocarcinoma or squamous cell) and tested the proposed signatures. The algorithm is shown in Figure 1.

## 3. Results

### 3.1. Survival Values at 2 Gy and Survival Curves of All Cell Lines

**Figure 2:**
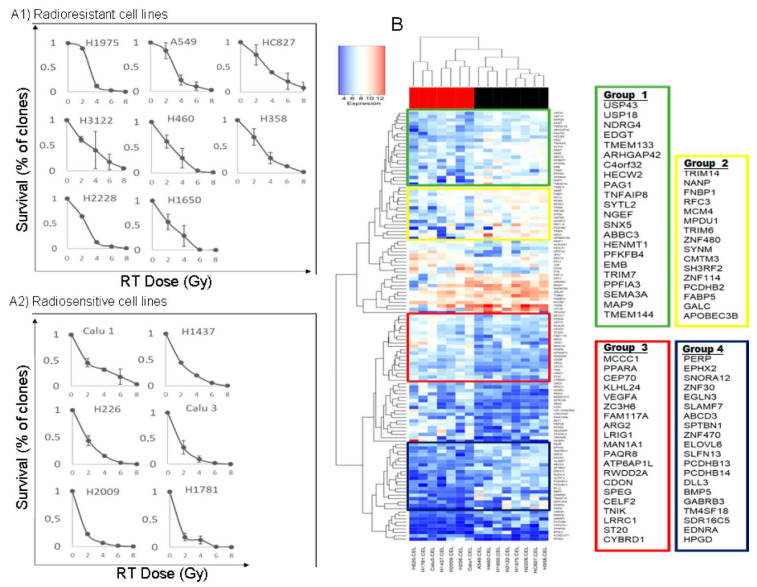
(**A**) Cell survival curves after radiation, measured by a clonogenicity assay. The ordinate axis represents the surviving fraction as a percentage, and the radiation dose administered (0–8 Gy) is represented on the abscissa axis. (**A1**) Radioresistant cell lines. (**A2**) radiosensitive cell lines. (**B**) Heatmap representing the result of the supervised clustering of the genes of interest. In the columns, the cell lines are presented (red represents the radiosensitive ones, and black represents the radioresistant ones), arranged according to the dendrogram located in the upper part of the figure. In the rows, the genes of interest are presented and grouped according to the dendrogram shown in the left part of the figure. See text for details.

We used the clonogenicity test to assess the response to ionizing radiation by determining the SF2 parameter (fraction surviving at 2 Gy) and counting the number of colony-forming units (CFU) at that dose. The mean value of the summation of all the SF2 Gy values of each of the cell lines was used to obtain a mean value (0.54), which we used as a cutoff point on which to establish a classification as sensitive lines (value of SF2 < 0.54) or resistant (SF2 ≥ 0.54) to radiation. This value, as well as the rest of the survival values at the different doses proposed (control, 4, 6, and 8 Gy), allowed us to generate the dose–response curves shown in Figure 2A. Table 1 shows the information on the SF values at 2 Gy of each cell line. The value of the H520 line was extracted from the literature (Table S2).

### 3.2. Identification of Genes with Differential Expression between Radiosensitive and Radioresistant Lines

To identify differentially expressed genes between radiation-resistant and radiation-sensitive lines, we employed a cDNA-based microarray analysis. Our panel of cell lines was mainly composed of the histological subtype of adenocarcinoma, but we also included squamous and large cell lineages. To analyze the differential transcriptional expression in the subset of radioresistant versus radiosensitive cells, we used a supervised grouping. We considered as a cutoff point those with |log2FC| > 1 and *p* < 0.05. We obtained a total of 127 genes with differential expression, of which 76 were overexpressed and 51 were underexpressed (Table S3). A heatmap was generated to represent the results (Figure 2B). We established four subgroups of genes with differential expression between sensitive and resistant lines (Figure 2B). In gene groups 1, 2, and 4, there was a trend toward higher expression levels in the lines considered radioresistant versus sensitive. In contrast, group 3 showed a trend toward higher levels of expression in radiosensitive lines.

### 3.3. Individual Validation of Genes with Differential Expression as Prognostic Biomarkers in the TCGA Cohort of Radiation-Treated Patients

We used the TCGA public cohort of patients with NSCLC (Pan Cancer Atlas) (2018 update) for the validation of our results. This cohort consisted of adenocarcinoma and squamous NSCLC. The main clinical criteria for the selection of patients were (1) having received radiotherapy treatment and (2) being classified as nonmetastatic at diagnosis. With these inclusion criteria, we selected a total of 107 patients.

Table S4 shows the clinical data of the selected patient cohort. The mean age at diagnosis was 63 years (range: 39–86 years). Fifty-nine of the patients were male, and 48 were female. The initial diagnosis of the patients and the treatments were carried out between 1992 and 2013. Regarding the pathological classification by stages, based on the TNM assessment (from the 3rd to the 7th edition, depending on the year of the diagnosis), 24/107 patients were classified as stage I, 33/107 patients as stage II, and 50/107 patients as stage III. Among the latter group, 42/107 patients were classified as stage IIIa, and 7 patients were classified as stage IIIb. Regarding the therapeutic approach, all patients received radiotherapy treatment, without being able to specify whether it was administered as an adjuvant or in the presurgical context or as a radical modality alone or in combination with systemic treatment. At the time of the cohort data collection (TCGA 2018 update), 57/107 patients had died and 68/107 patients had experienced progression of their disease. The median PFS, cancer-specific survival (CSS), and OS were 22.9 months (range: 0.72–140), 41.6 months (range: 0.72–140), and 32.7 months (range: 0.72–140), respectively (Figure S1). OS, PFS, and CSS at 1, 2, and 5 years were 80%, 66%, and 33%; 67.5%, 50%, and 21%; and 82.5%, 72.5%, and 44%, respectively.

To determine whether there are factors that affect OS and PFS, a series of clinical and pathological variables were considered to analyze their influence on these parameters. To facilitate the statistical analysis, the variables were dichotomized. These variables were sex (male versus female); age at diagnosis (less than or greater than 63 years); year of diagnosis (before or after 2008); histological subtype (adenocarcinoma versus squamous cell); T component (T1 and T2 versus T3 and T4); nodal load (n0 and n1 versus n2 and n3), and tumor stage (stage I and II versus stage III). Likewise, the gene signature was dichotomized into low- and high-risk patients based on whether the value of the individual signature was less than or greater than/equal to the median of the values of the established signature. The information regarding the estimation of OS and PFS as a function of the variables described above is shown in Table S5. A Kaplan–Meier analysis was used, and the curves were compared by means of the logarithmic rank test, considering a *p*-value of <0.05 as statistically significant. As shown, only patients with a high-risk versus a low-risk gene signature showed statistically significant differences in OS. Regarding PFS, the histological adenocarcinoma subtype and the proposed high-risk gene signature were statistically significantly associated with worse PFS. From the bioinformatics exploration of the results obtained considering all cell lines, a total of 127 genes were obtained, with differential expression between radiosensitive and radioresistant lines (absolute value of log2FC > 1 and adjusted *p*-value < 0.05) (Table S3). When analyzing the RNAseq data of the 127 genes reflected above for the selected TCGA cohort, we identified a lack of information for six of them (C4orf32, KIAA1324L, LINC00597, LOC100287896, SELENOP, and TMEM133). Thus, considering the expression levels of the 121 remaining genes, a survival analysis was carried out using univariate Cox regression, through which we obtained a total of 21 genes whose expression levels were significantly related to OS in the selected patient cohort (Table S6).

**Figure 3:**
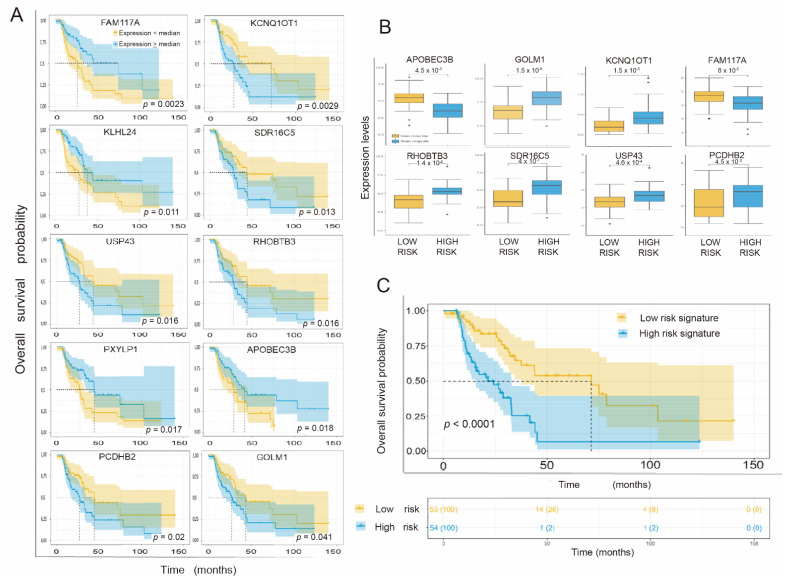
(**A**) Kaplan–Meier curves of the overall survival probability of the patients according to stratification by the expression levels of each individual gene. The information regarding the 10 genes of interest (see text) is ordered by level of statistical significance (*p*-value). The ordinate axis represents the probability of survival (0–1), and the abscissa axis represents the overall survival in months. Yellow represents patients whose expression level of the gene in question was lower than the median expression of the gene, and blue represents patients with expression levels equal to or greater than the median expression of the gene in question. (**B**). Box plot showing the expression levels (logTPM) (normalized) of each gene that makes up the signature based on the prognostic group (low and high risk). (**C**). Kaplan–Meier curves in which the overall survival probability of the patients is represented according to their grouping according to the established cutoff point, which corresponds to the median of the risk scores of each patient in the cohort, separating the latter into two groups (high or low risk, depending on whether their individual risk score is higher than/equal to or lower than the cutoff point, respectively). Likewise, the number of patients at risk in each group is represented according to the established time intervals. *, *p* < 0.05; **, *p* < 0.01.

Focusing on the previously exposed genes, we carried out an analysis of survival estimation using Kaplan–Meier curves, taking the median gene expression as the cutoff point to divide the samples into high and low expression levels. A total of 10 genes of the 21 identified above yielded a value of *p* < 0.05 by the log-rank test (Figure 3A). Thus, the genes FAM117A, KCNQ1OT1, KLHL24, SDR16C5, USP43, RHOBTB3, PXYLP1, APOBEC3B, PCDHB2, and GOLM1 achieved statistical significance in the univariate Cox regression analysis and in the OS estimation by Kaplan–Meier and SPL (PFS) by Kaplan–Meier.

### 3.4. Obtaining the Prognostic Signature

We selected the 10 genes identified in the previous section as potential candidates to form part of the prognostic signature. We then performed a multiple Cox regression with a three-iteration cross-validation in which 2/3 of the data (71 patients) were used as the training cohort and 1/3 (36 patients) were used as the trial cohort. We also applied Lasso regularization (L1 type) to the regression to select the genes that influence survival in a more significant way. We obtained coefficients (betas in the Cox regression) for the genes APOBEC3B, GOLM1, FAM117A, KCNQ1OT1, SDR16C5, PCDHB2, RHOBTB3, and USP43. Next, we obtained a signature value for each of the patients, starting from the expression values (logTPM) of each gene of interest multiplied by their respective coefficients. Finally, we performed a univariate Cox regression analysis for all values of the firm (risk scores) as a result of the linear combination of the regression coefficient multiplied by the expression level of each gene in question:

Risk Score = (−0.1353493) × Apobec3B expression + 0.0671513 × Golm1 expression + (−0.4002509) × Fam117A expression + 0.6455878 × KCNQ1OT1 expression + 0.0613337 × SDR16C5 expression + 0.0542689 × PCDHB2 expression + 0.0309122 × RHOBTB3 expression + 0.0834290 × USP43 expression.

The expression of the gene in each group is shown by the box diagram in Figure 3B. All genes showed a statistically significant differential expression level depending on their relationship with each risk group.

Using a univariate Cox regression analysis of the prognostic signature, patients associated with the adverse prognostic group showed a significantly poorer OS than those associated with the favorable group, with an HR of 3.9 (95% CI 2.39–6.37) and a *p* < 0.0001. The concordance index was 0.713 ± 0.0374. The survival prediction analysis based on the proposed gene signature using ROC curves at 12, 24, and 60 months yielded AUC values of 0.73, 0.72, and 0.79, respectively (Figure S2A). For the Kaplan–Meier analysis (Figure 3C), we considered the median of the risk scores as the cutoff point to separate the two prognostic groups. This value is −0.8. The patients whose gene signature showed expression levels lower than the cutoff point and who had better survival are identified in yellow (median OS of 71.47 months (95% CI 36.7–106.2)), and the patients in which the signature values were equal to or above said point and had a worse prognosis are shown in blue (median OS of 24.13 months (95% CI 11.98–36.27)).

### 3.5. Individual Validation of Genes with Differential Expression between Radiosensitive and Radioresistant Lines as a Predictive Biomarker of Response in the TCGA Cohort of Interest

**Figure 4:**
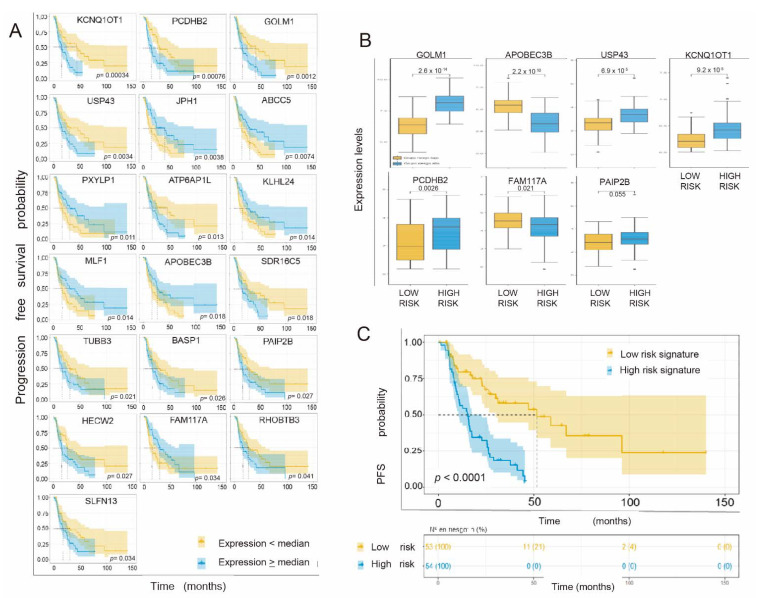
(**A**). Kaplan–Meier curves with the progression-free survival (PFS) probability of the 19 individual genes of interest (*p*-value < 0.05) ordered by level of statistical significance. The probability of PFS (0–1) is represented on the ordinate axis and the PFS in months on the abscissa axis. Yellow represents patients whose expression level of the gene in question was lower than the median, and blue represents patients with expression levels equal to or greater than the median. (**B**). Box diagram showing the expression levels (logTPM) (normalized) on the ordinate axis of each gene as a function of each prognostic group (low or high risk). (**C**). Kaplan–Meier curves of the probability of PFS of the patients according to their signature by the established cutoff point, which corresponds to the median of the risk scores of each patient in the cohort, separating the latter into two groups (high or low risk, depending on whether their individual risk score is higher than/equal to or lower than the cutoff point, respectively). Likewise, the number of patients at risk in each group is represented according to the established time intervals. *, *p* < 0.05; **, *p* < 0.01.

We considered PFS as a variable for evaluating the effect of cancer treatments in our cohort of interest. As in the OS section, we considered the 127 differentially expressed genes and identified 121 of them in the RNA-seq data from our TCGA cohort. We performed an impact analysis on PFS using the univariate Cox regression test. Table S7 shows the 29 genes whose impact on PFS was statistically significant. Focusing on the previously uncovered genes, we carried out a predictive analysis of SLP using Kaplan–Meier curves, taking the median of the expression of the gene as a cutoff point to divide the samples into high expression levels and low expression levels. Survival curves with *p* < 0.05 are shown in Figure 4A.

Thus, the genes KCNQ1OT1, PCDHB2, GOLM1, USP43, JPH1, ABCC5, PXYLP1, ATP6AP1L, KLHL24, MLF1, APOBEC3B, SDR16C5, TUBB3, BASP1, PAIP2B, HECW2, FAM13B3A, FAM117A, and RHFAM137A individually showed a statistically significant impact on PFS by Cox regression analysis and maintained statistical significance in the estimation of PFS by Kaplan–Meier analysis. We selected these genes to develop the predictive response signature. We carried out a multivariate Cox regression analysis with L1-type regularization with cross-validation of three iterations. Only 7 out of 19 genes were kept in the model summarized in the following formula:

Risk score = (−0.1462772) × APOBEC3B expression + 0.1824914 × GOLM1 expression + (−0.1364778) × FAM117A expression + 0.5059698 × KCNQ1OT1 expression + 0.0545114 × PAIP2B expression + 0.0624953 × PCDHB2 expression + 0.0317391 × USP43 expression.

We calculated the risk score based on the seven genes of interest in each patient, and then, using the statistical package Survminer R, we identified the cutoff point for the risk score, classifying all patients in a high- or low-risk cohort based on the established cutoff point. The expression values of each gene in each of the two differentiated groups are shown in Figure 4B by means of a box diagram. All the genes of the signature except PAIP2B showed a statistically significant differential expression level depending on their relationship with each prognostic group (Mann–Whitney U test with *p* < 0.05).

Next, a univariate Cox regression analysis of the predictive signature was carried out, obtaining an HR value of 5.04 (95% CI 3–8.47) and *p* < 0.001. The concordance index was 0.703. Finally, we carried out a prediction analysis of PFS based on the proposed gene signature. To do this, we performed an analysis using ROC curves at 12, 24, and 60 months (and subsequently a Kaplan–Meier analysis, considering the median Figure S2B) of the risk scores as the cutoff point, whose value was +0.72, to separate the two prognostic groups (Figure 4C). Patients whose gene signature showed expression levels lower or higher than this cutoff point obtained a median PFS of 51.55 (95% CI 20.1–83) and 15.45 months (95% CI 10.1–20.8), respectively.

### 3.6. Generation of a Common Gene Signature for Global Survival Prediction and Progression-Free Survival

After obtaining the gene signatures with prognostic and predictive response values, we observed that six gene elements were shared between them (APOBEC3B, GOLM1, FAM117A, KCNQ1OT1, PCDHB2, and USP43), which led us to the generation of a single signature with prognostic ability for OS and PFS. In this case, using the OS data of the cohort, we obtained a risk score summarized in the following formula:

Risk score **=** (−0.1783969) × APOBEC3B expression + 0.0877709 × GOLM1 expression + (−0.4162868) × FAM117A expression + 0.8842986 × KCNQ1OT1 expression + 0. 0859888 × PCDHB2 + 0.1117924 × USP43 expression.

**Figure 5:**
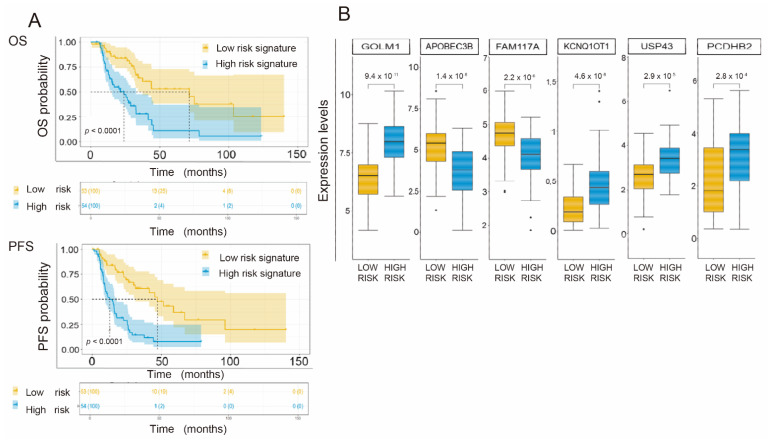
(**A**). Common signature of six genes for OS and PFS. Kaplan–Meier curves in which the probability of OS (OS) and PFS (PFS) is presented as a function of the risk group of the proposed firm (low or high risk). (**B**). Box plot showing the expression levels (logTPM) on the ordinate axis of each gene as a function of each prognostic group (low or high risk). *, *p* < 0.05; **, *p* < 0.01.

We then performed a signature univariate Cox regression analysis for OS and PFS of total NSCLC. As reflected in Table S8, the HRs for OS and PFS associated with the high-risk gene signature were 3.23 and 3.46, with concordance indices of 0.71 and 0.72, respectively. We carried out a prediction capacity analysis of the common signature using ROC curves, with AUCs at 12, 24, and 60 months for OS of 0.69, 0.70 and 0.81 and for PFS of 0.67, 0.78, and 0.79, respectively. For the Kaplan–Meier curves, we established a cutoff point based on the median of the firm’s values (risk score), whose value turned out to be −1.059, and obtained two curves compared by means of the log-rank test. The ROC curves are shown in Figure S3, and the Kaplan–Meier curves are shown in Figure 5A. Finally, we determined the expression levels of each gene based on the risk group considered in the signature (low or high risk). We compared these levels using the Mann–Whitney U test and presented them as box plots (Figure 5B).

### 3.7. Application of the Gene Signature for Prediction of OS and PFS Depending on the Histological Subtype

Given that the proposed gene signature was generated without distinction by main histological subgroup (adenocarcinoma versus squamous cell), we proposed checking whether the signature was valid for each subtype individually. We proceeded methodologically in the same manner as in the previous sections for the application of the signature. Table S8 shows the values of the univariate Cox regression analysis of the common gene signature, distinguishing between adenocarcinoma and squamous cell carcinoma. When comparing the signature values with those obtained in the previous analysis without distinction by histological subtype, we observed similar HR values between them, without losing statistical power.

**Figure 6:**
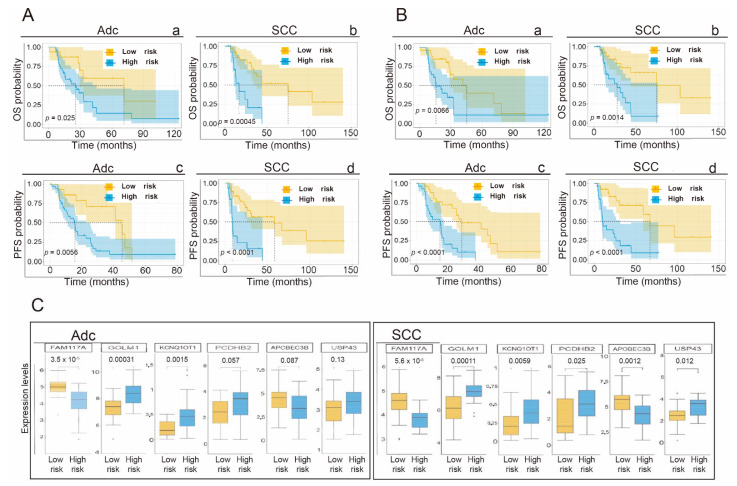
(**A**) Kaplan–Meier curves in which the probabilities of OS and PFS are represented as a function of the risk group associated with the gene signature for the subgroup of patients with adenocarcinoma, Adc, (a and b) and for the squamous, SCC, subtype (c and d). Each graph shows the number of patients at risk for different times. The value obtained from the analysis of the global cohort was used as a cutoff point to separate both groups, SCC and Adc. (**B**) Kaplan–Meier curves in which the probabilities of OS and PFS are represented as a function of the risk group associated with the gene signature for the subgroup of patients with adenocarcinoma, Adc, (a and b) and for the squamous, SCC, subtype (c and d). Each graph shows the number of patients at risk for different times. The median of the risk score values of each cohort, Adc or SCC, was independently used as a cutoff point to separate each respective group. (**C**) Box diagram showing the expression levels (logTPM) on the ordinate axis of each gene that makes up the gene signature according to the prognostic group (low or high risk). The results of the adenocarcinoma cohort analysis (a) and squamous cell carcinoma analysis (b) and the gene elements are ordered from left to right according to the *p*-value (Mann–Whitney U test). *, *p* < 0.05; **, *p* < 0.01.

The ROC curves with the AUC values at 12, 24, and 60 months are shown in Figure S4. We appreciate how the predictive capacity of the signature is maintained in both adenocarcinoma and squamous cell carcinoma (AUC values equal to or greater than 0.6 in all cases). Figure 6A shows the Kaplan–Meier curves, with statistically significant separation (log-rank test *p* < 0.05) between the patients assigned to the low- or high-risk group, separated according to the cutoff point used in the global cohort (−1.05).

We also decided to carry out an additional Kaplan–Meier analysis, considering as the cutoff point the median value of the risk scores obtained independently in the adenocarcinoma or squamous carcinoma subsets. Thus, the cutoff points, when considering the median, for the adenocarcinoma cohort and squamous cancer cohort were −0.81 and −1.48, respectively. The KM curves are shown in Figure 6B. The differences in survival estimates for high- and low-risk patients are still statistically significant. Finally, we determined the expression levels of each gene based on the risk group considered in the signature (low or high risk). We compared these levels using the Mann–Whitney U test and presented them as box plots (Figure 6C).

### 3.8. Univariate and Multivariate Analysis of Clinical and Pathological Variables, Their Impact on Overall Survival and PFS and Their Influence on the Proposed Gene Signature

To verify the potential impact of the variables considered on the OS of the patients, we first performed univariate Cox regression analysis, followed by a bivariate analysis to identify the influence of these variables on the impact of the gene signature on OS. In the univariate Cox regression analysis, only the gene signature proposed in our work showed a statistically significant impact on OS, with an HR for the high-risk group of 2.99 (95% CI for HR of 1.72–5.19) and *p* < 0.0001 (Table 2). The variable “tumor stage,” and, within it, stages I and II, was related to a decrease in the risk of death (HR = 0.60 with 95% CI for HR of 0.35–1.01), with a trend toward statistical significance (*p* = 0.051).

We ruled out the need for a multivariate analysis after performing a bivariate analysis (File S1) where the variable “prognostic gene signature” was established as the main one. None of the variables considered had a statistically significant impact on survival or modified the impact of the prognostic gene signature in the selected cohort.

## 4. Discussion

NSCLC, which encompasses the most frequent histological subtypes of lung cancer, is a neoplastic entity whose biological behavior is highly heterogeneous. Molecular heterogeneity could explain the different responses and behaviors before cancer treatments, as well as the impact of the neoplasm on the OS of patients. Considering this molecular heterogeneity, we believe that the identification of gene signatures with prognostic and predictive capacity of response to cancer treatment, in this case, focused on radiotherapy treatment, could be of great help in optimizing the therapeutic approach for patients.

Currently, the tumor staging system by TNM classification continues to be the most powerful instrument for predicting patient survival and so is a focus for the oncology community in terms of therapeutic approaches in the case of NSCLC and most neoplasms [2,61,62]. Despite efforts to obtain clinical, pathological, and/or molecular information that could serve to predict responses to treatment and improve prognostic capacity, there are currently no validated biomarkers in NSCLC that facilitate the oncology community’s decisions regarding individualized treatment selection in the nonmetastatic setting. There are multiple proposals for gene signatures that attempt to predict survival or response to treatment (not radiotherapy], but these mainly focus on the early stages or metastatic stage at diagnosis. In contrast, there are few studies that propose such predictive and/or prognostic elements in locally advanced nonmetastatic stages, where treatment with ionizing radiation plays an important role. The radiation oncologist lacks molecular markers that serve to guide the radiotherapy treatment to be used, beyond the general recommendations derived, for example, from the pathological report, considering the status of the surgical margins or positive nodal load, among others [2,61]. One of the difficulties we face when we propose the identification of predictive and prognostic signatures in NSCLC is an inability to identify whether the clinical, therapeutic, histological, or molecular variables have the same weight when determining the sustained therapeutic response and global survival [63].

The main objective of our study was to develop and validate gene signatures with prognostic and predictive capacity for response to radiotherapy. We found a single signature that was valid as a prognostic element and as a predictor of therapeutic response with six gene elements (APOBEC3B, GOLM1, FAM117A, KCNQ1OT1, PCDHB2 and USP43, File S1). The HR values for OS and PFS associated with the high-risk gene signature were 3.23 and 3.46, with concordance indices of 0.71 and 0.72, respectively (*p* < 0.0001). In the case of the adenocarcinoma subgroup, the HR of the high-risk gene signature for OS and PFS was 3.52 and 3.76, respectively (*p*-value < 0.001). In the case of SCC, the values of the signature’s HR for OS and PFS were 3.87 and 3.78, respectively. These results indicate the value of the proposed gene signature regardless of the main histological subtype.

There are several signatures reported to be diagnostic or prognostic for lung cancer in the literature [23,45,46,64,65,66] that were obtained by different methods, cohort types, or statistical strategies (File S1. In all cases, the proposed genes that make up the signatures do not coincide among them or with ours, probably due to notable differences in the methodology and materials used in these studies and in ours, differences in the patient cohorts, or the fact that most of the patients did not receive adjuvant treatment, unlike the cohort used in our study.

In contrast to the works discussed above, articles published on the identification of predictive or prognostic gene markers focused on cohorts whose main treatment was ionizing radiation are scarcer and practically nonexistent in the specific case of NSCLC. Torres-Roca and collaborators [67] identified genetic elements common to all neoplasms, which could explain the differences in radiosensitivity observed both in vitro and in clinical practice. The generation of the so-called “radiosensitivity index” (RSI) forms a predictive signature of response to radiotherapy treatment composed of the AR, cJUN, STAT1, PKC, RELA, ABCc, SUMO1, CDK1, HDAC1, and IRF1 genes, which has been subsequently validated in cohorts of patients with breast cancer, head and neck cancer, esophageal cancer, rectal cancer, and glioblastoma multiforme [50,67,68]. The work by Scott and collaborators [56] proposes a model to adapt the radiotherapy prescription to the individual sensitivity of the tumor of each patient. The model, called GARD (genome-based model for adjusting radiotherapy dose), combines the information derived from the radiosensitivity index (RSI) and the linear quadratic model (LQ model), which proposes the existence of two parameters that impact the cytotoxic capacity of radiation, one of them being proportional to the dose of radiation administered (factor α) and the other being proportional to the square of the dose (factor β). This mathematical model has been used for decades to calculate the equivalent biological dose of different radiotherapy treatment schemes, taking into account the α/β index of each tumor, which has been used to propose altered radiotherapy fractionations that have allowed the attainment of biologically equal or superior results to the treatments based on daily normal fractionation (1.8/2 Gy/fraction) [69,70]. This work uses multiple cohorts of different neoplastic entities (breast, esophageal, head and neck, stomach, cervical, glioma, pancreatic, lung, and melanoma and nonmelanoma skin cancers) and establishes a numerical value for GARD (usually in the range of 1–200), with a higher GARD level being related to a greater therapeutic effect of radiotherapy treatment and vice versa. The authors emphasize that the GARD model is not useful for predicting survival. However, they insist that different GARD values adequately predict the differences in therapeutic response (to ionizing radiation) seen in daily clinical practice between the different tumor subtypes or different affected anatomical locations treated, which could be related to the impact in terms of locoregional control of the disease, as well as to patient survival. One of the potential criticisms of this extensive work is the presumption that the tumor response after ionizing radiation depends predominantly on the tumor biology itself, without considering other nontumor factors per se (tumor environment, comorbidities, homeostatic, immunological, etc.) or without considering the usual combination with other oncological treatments, which is usually the norm in the multidisciplinary approach of most of the neoplasms considered in the study. Based on the RSI and GARD models, more recent works continue to propose their application in routine clinical practice with the aim of predicting the response to ablative stereotaxic or normofractionated treatment at the lung or other locations and the adaptation of fractionation and DBE in those histological lines that usually show a worse response to ionizing radiation [71,72]. There are no strict genetic/molecular factors, such as the general condition of the patient, tumor size, nodal load, age, existence of comorbidities, previous treatments administered and TNM classification, or other elements identified in samples (markers of inflammation such as interleukins and C-reactive protein; indirect markers of hypoxia such as osteopontin, carbonic anhydrase IX and lactate dehydrogenase; or indirect markers of tumor burden such as carcinoembryonic antigen or cytokeratin 21–1 fragments) that have been used classically to predict the response to treatment and the vital prognosis, both in lung cancer and in other solid and hematological neoplasms [73,74,75].

These factors, generally validated in univariate studies and with significant biases, were considered in the work published by Dehing-Oberije et al., who proposed a combination of clinical factors (the WHO health status classification (WHO-PS), forced expiratory volume in the first second (FEV1), gross tumor volume (GTV) equivalent to the size of the main tumor component, nodal load, and sex) with biomarkers obtained from peripheral blood (CEA and IL-6), thus obtaining an improvement in the prognostic capacity at two years of patients affected and treated for NSCLC [60]. In our study, centered on a cohort of 107 TCGA patients, no variable considered had a statistically significant impact on OS in the univariate analysis by Cox regression. In the bivariate analysis, considering the gene signature as the main variable, none of the variables modified the HR value associated with the signature. In the case of the impact of the variables considered on PFS, only the variable “histological adenocarcinoma subtype” was statistically significant in the univariate analysis. However, in the bivariate analysis, the histological subtype lost statistical significance and did not substantially modify the HR value associated with the gene signature.

In our work, we have proposed a basic approach with potential translational capacity, although there are several limitations: (1) The discovery set we used was a total of 15 commercial NSCLC lines, which may limit the statistical power during the bioinformatic analysis. (2) We assessed the response to ionizing radiation with the clonogenicity test, as this is the standard for the determination of survival in Rt. As reflected in the literature [51,76,77,78], there are some differences in the published SF2 values of the different cell lines, which implies that there may be possible small differences when establishing the classification of radiosensitive and radioresistant. (3) Most of the publicly available bioinformatics information databases on NSCLC contain samples of patients mainly in localized and/or metastatic stages, which generally have not received or do not reflect information on radiotherapy treatment, which has greatly limited the sample size used in our study (*n* = 107), as well as additional cohorts for the validation of our signatures.

The generation of gene signatures of a prognostic and/or predictive nature of response to some treatment does not usually assess other biological factors not directly related to the biology of the tumor itself. These factors, whose genetic and epigenetic bases can condition the response to certain cancer treatments and even significantly condition the overall survival of patients, are undoubtedly the greatest biases when giving translational value to these signatures. In our study, we carried out a univariate analysis of multiple clinical and pathological variables, and a bivariate analysis by Cox regression without a factor will substantially modify the HR value of the proposed gene signature.

The generation of new gene signatures with prognostic and/or predictive capacity in pathologies such as NSCLC can significantly benefit patients. In our study, we proposed a prognostic gene signature for OS with the ability to predict PFS. Knowledge about the respondent profile or the vital prognosis of the patient prior to the start of treatment can help us optimize the therapeutic approach and avoid dreaded and frequent iatrogenic events. However, our study still needs additional validation steps before clinical use. For example, validation of a reference cutoff against which to compare each patient, such as their blood, nontumor lung tissue, or a pool of tumor biopsies, is needed, remembering that our signature of high and low risk for each gene was generated based on the median of a cohort of tumor samples. Additionally, and very importantly, it would be necessary to calibrate the relative contribution of each of the genes to the predictive signature and organize a prioritization algorithm for these genes. All of this could be part of future work. We believe that our work is a good example of how it is possible to perform translational radio-oncology, and we trust that, together with the results already published and future work, we can contribute to achieving the final objective of the entire oncology community, which is to improve the quality of life and prognosis of cancer patients.

## 5. Conclusions

The oncology community needs more tools and knowledge to improve the ability to predict response to cancer treatments and patient survival. This is transcendental in such incidental and fatal neoplasms as NSCLC. In this work, the characterization of the differential gene expression profile based on the radiophenotype in NSCLC cell lines allowed us to obtain a gene expression signature with prognostic capacity and therapeutic response prediction. This gene expression signature was further validated in a cohort of patients diagnosed in early and locally advanced stages and who had received radiotherapy among other treatments. We believe, pending its usefulness in the clinic, that this biological approach and a consideration of the treatment used is key to the potential identification of new biomarkers that may lead us to improve oncological results in this or other neoplasms.

## Figures and Tables

**Table 1:** Values of the surviving fraction at 2 Gy (SF2) of all cell lines. Results of clonogenicity tests.

Cell Line	SF2	SD
Radioresistant		
H1975	0.891	0.0104
A549	0.832	0.162
HC827	0.745	0.2
H358	0.676	-
H2228	0.646	0.056
H3122	0.622	0.056
H460	0.617	0.129
H1650	0.570	0.156
Radiosensitive		
Calu1	0.454	0.079
H520	0.490	-
H1437	0.448	-
H226	0.430	0.09
Calu3	0.325	0.131
H2009	0.228	0.024
H1781	0.180	0.067

**Table 2:** Univariate Cox regression analysis. The impact of independent categorical variables on OS was analyzed.

Variables	Beta	ET	Wald	HR	95% CI for HR		*p*-Value
					Inferior	Superior	
Age at diagnosis	−0.25	0.27	0.88	0.78	0.46	1.32	0.35
Year of diagnosis	0.46	0.31	2.28	1.59	0.87	2.91	0.13
Sex	−0.21	0.27	0.65	0.81	0.48	1.36	0.42
Histological subtype	0.38	0.27	1.97	1.46	0.86	2.48	0.16
T component	−0.38	0.29	1.71	0.68	0.39	1.21	0.19
Nodal load	−0.24	0.27	0.80	0.78	0.46	1.34	0.37
Tumoral stage (AJCC)	−0.52	0.27	3.73	0.60	0.35	1.01	0.05
Genetic signature OS	1.10	0.28	15.16	2.99	1.72	5.19	<0.0001

CI = confidence interval; HR = hazard ratio.

## Data Availability

Dataset for transcriptional analysis of cell lines (code: GSE197109) was stored at GEO. Additional datasets used in this study are public; references and code can be consulted in the text.

## Supplementary Materials

The following are available online at https://www.mdpi.com/article/10.3390/cancers14092054/s1, Table S1: Histological information and the molecular profile of some relevant NSCLC mutations of the cell lines used in this study. Information on the histology and molecular profile of relevant genes in NSCLC. CBA: bronchioloalveolar carcinoma, CCG: large cell carcinoma. WT: wild type (native). MUT: mutated. Table S2: SF2 value obtained from the scientific literature, Table S3: List of differentially expressed genes between radiosensitive and radioresistant cell lines, Table S4: Summary of the characteristics of the 107 patients in the main study cohort, Table S5: Results of the univariate analysis (log rank test) for potential prognostic and predictive factors of response, Table S6: Genes with a significant impact (p < 0.05) on overall survival measured by univariate Cox regression. They are shown in descending order by p-value, Table S7: Genes with a significant impact (p < 0.05) on PFS measured by univariate Cox regression. Genes are sorted in descending order by p-value, Table S8: Results of the analysis of the common gene signature by univariate Cox regression for OS and PFS, Table S9: Bivariate Cox regression analysis. Assessment of the effect of independent categorical variables on the HR of the prognostic gene signature of OS, Table S10: Univariate Cox regression analysis. Impact of the independent categorical variables on the PFS, Table S11: Bivariate Cox regression analysis. Assessment of the effect of independent categorical variables on the HR of the prognostic gene signature of PFS, Figure S1: Kaplan–Meier curves showing progression-free survival (PFS), cancer-specific survival (SCE), and overall survival (OS) of the cohort of interest, Figure S2: (A) ROC curves as a function of time (T). AUC: area under the curve. OS; (B) ROC curves in function of time (T). AUC: area under the curve. PFS: progression free survival, Figure S3: ROC curves at 12, 24, and 60 months of the common gene signature for OS (a) and PFS (b), Figure S4: ROC curves at 12, 24, and 60 months of the common gene signature for OS (a and c) and PFS (b and d), according to the histologic group adenocarcinoma or squamous cell carcinoma (SCC). File S1: Supplementary information.
