# Supplementary material for: A Six-Gene Prognostic and Predictive Radiotherapy-Based Signature for Early and Locally Advanced Stages in Non-Small-Cell Lung Cancer
Source: Cancers (Basel). 2022 Apr 19;14(9):2054. doi: 10.3390/cancers14092054 (PMC9099638; doi:10.3390/cancers14092054)
Supplement: Supplementary file 1 [file cancers-14-02054-s001.zip › cancers-1678074-supplementary-File S1.pdf]

cancers MDPI

Supplementary information 1

Before ruling out the need for a multivariate analysis, we wanted to realize a bivariate analysis, where the variable "prognostic gene signature" was established as the main one. **Supplementary Table S9** shows the results of this analysis. None of the variables considered had a statistically significant impact on survival or modified the impact of the prognostic gene signature in the selected cohort. As an example, in the case of bivariate analysis with the variable "tumor stage", the HR associated with the variable "prognostic signature" (2.81), indicates that patients with a high-risk prognostic signature have a risk of death 2.81 times greater than those with a low-risk gene signature, once the possible influence of tumor stage on OS is controlled. Furthermore, the deleterious effect that the unfavorable prognostic signature has on OS is statistically significant (p value <0.0001), when the effect of tumor stage on OS is controlled. As the HR of the variable "gene signature" of the present model (2.81) is very similar to that obtained in the univariate model (2.99), tumor stage is not a confounding factor in the relationship between prognostic gene signature and OS, and neither it is a risk factor for OS since its HR is not statistically significant (p-value = 0.24). This interpretation is extensible to the rest of the variables included and allows us to omit a subsequent multivariate analysis.

As in the OS section, we next realized a univariate Cox regression analysis of each of the clinical and pathological variables considered to determine their impact on PFS. As shown in **Supplementary Table S10**, the histological subtype adenocarcinoma and the high-risk gene signature were found to impact PFS with an HR of 1.74 and 3.66 and a p value of 0.03 and <0.0001, respectively. In the bivariate analysis by Cox regression, taking the predictive gene signature as the main variable and confronting it with the rest of the variables, none turned out to be a statistically significant modifying or confounding factor in the impact of the gene signature on PFS **(Supplementary Table S11)**.

Supplementary information 2

APOBEC3B (apolipoprotein B mRNA editing enzyme catalytic subunit 3B): This gene is a member of the cytidine deaminase family. It is located on the long arm of chromosome 22 (22q13.1). These proteins are thought to be RNA editing enzymes and play an important role in cell growth or cell cycle control, among other functions. It has been suggested that the deletion of this gene could be related to an increased susceptibility to develop neoplasms. GOLM1 (golgi membrane protein 1): codes for a type 2 transmembrane protein of the Golgi apparatus. The gene is located on the long arm of chromosome 9 (9q21.33). Numerous publications show the relationship of altered GOLM1 expression levels with proliferation, migration, invasion and inhibition of apoptosis in prostate, breast, pancreas cancer, hepatocellular carcinoma and squamous cell carcinoma of the oral cavity. Likewise, and related to NSCLC, Liu et al. Published an article in which they relate the overexpression of GOLM1 with detrimental overall survival and recurrence-free survival in patients with the adenocarcinoma subtype. They were based on data from the TCGA lung cancer cohort.

FAM117A (family with sequence similarity 117 member A): is a gene that codes for the FAM117A protein. The gene is located on the long arm of chromosome 17 (17q21.33). We have not identified relevant information about its involvement in cancer or specifically in NSCLC. KCNQ1OT1 (KCNQ1 opposite strand / antisense transcript 1): the gene is epigenetically regulated. It is located on the short arm of chromosome 11 (11p15.5). The transcript interacts with chromatin and regulates the transcription of multiple genes through epigenetic modifications. It appears to play an important role in colorectal carcinogenesis and a role has been suggested as a facilitator of progression in NSCLC138. PCDHB2 (protocadherin beta 2): is a gene that codes for a protocadherin that is part of the subset of beta protocadherins that are located on the long arm of chromosome 5 (5q31.3). These neural lineage cadherin-type proteins are part of the plasma membrane, and although their exact

function is unknown, they appear to play an important role in cell-cell junction in melanocytes and melanoma. USP43 (ubiquitin specific peptidase 43): codes for a protein from the family of deubiquitinizing proteases. The gene is located on the small arm of chromosome 17 (17p13.1). Some studies have related the expression levels of the gene with the regulation of the cycle and the epithelial-mesenchymal transition process in breast cancer. We have not found any references regarding NSCLC.

Supplementary information 3

Beer et al., 64. propose a gene profile composed of 50 genes, which allows the identification of a subgroup of patients within those classified as stage I whose behavior and survival are similar to those classified as stage III. Tomida et al. Proposed a prognostic signature with 25 genes resulting from the analysis of 50 patients undergoing surgery for NSCLC with high subtype heterogeneity 65. Raponi et al. 32 used a total of 129 SCC samples (the majority from stage I disease patients) and performed a microarray analysis with validation by RT-PCR and immunohistochemistry. They obtain a group of 50 genes with the ability to separate the SCC tumors by prognostic subgroups. Lau et al. Published a prognostic signature of 3 genes, STX1A, CCR7 and HIF1A, focused on NSCLC in early stages 66. A prognostic signature of 5 genes (DUSP6, MMD, STAT1, ERBB3 and LCK), was published by Chen and cols. which were the result of a combined analysis of microarrays and RT-PCR in tumor samples from 101 patients who underwent surgery (mix of adenocarcinoma, SCC and other subtypes) 67. Subsequently, the results were validated in an independent cohort of 60 patients and in a microarray set of 86 patients. In the multivariate analysis with other clinical variables such as age or stage, the signature maintained its statistical significance. Zuo et al. Propose a 6-gene signature with the ability to predict disease-free survival and overall survival in NSCLC, without discriminating by histological subtypes 68. They were based on the combination of genetic information from 3 public databases that encompass all histological subtypes (but mostly adenocarcinomas). After obtaining the candidate genes with prognostic capability, a validation was carried out with the TCGA lung cancer cohort. Later, they proposed an 8-gene prognostic signature for patients with early-stage NSCLC 69.
