# Supplementary material for: A Six-Gene Prognostic and Predictive Radiotherapy-Based Signature for Early and Locally Advanced Stages in Non-Small-Cell Lung Cancer
Source: Cancers (Basel). 2022 Apr 19;14(9):2054. doi: 10.3390/cancers14092054 (PMC9099638; doi:10.3390/cancers14092054)
Supplement: Supplementary file 1 [file cancers-14-02054-s001.zip › cancers-1678074-supplementary.pdf]

cancers MDPI

Table S1. Histological information and the molecular profile of some relevant NSCLC mutations of the cell lines used in this study. Information on the histology and molecular profile of relevant genes in NSCLC. CBA: bronchioloalveolar carcinoma, CCG: large cell carcinoma. WT: wild type (native). MUT: mutated.

Cell line	Histology	EGFR	KRAS	Others
A549	Adenocarcinoma	WT	WT	
H1650	CBA	MUT (exon 19)	WT	
H1975	Adenocarcinoma	MUT (790M)	WT	CDKN2A/Pi3KCA/TP53
H2009	Adenocarcinoma	WT	MT	
H358	CBA	WT	MT	
H460	CCG	WT	MT	
H520	SCC	WT	nd	
HCC827	Adenocarcinoma	MUT	WT	
H1437	Adenocarcinoma			CDKN2A/TP53
H1781	Adenocarcinoma			
H2228	Adenocarcinoma			ALK
H226	SCC			
Calu 1	SCC		MT	
Calu 3	Adenocarcinoma	WT	MT	Amplification ERBB2 /p53/CDKN2A
H3122	Adenocarcinoma			ALK

Table S2. SF2 value obtained from the scientific literature.

Cell line	SF2 value found in the scientific literature								
	Carmichael 1989	Eschrich 2009	Tumati 2013	Abazeed 2014	Ahn 2014	Anakura 2019	Jung 2019	Girdhani 2019	Our SF2 results
H1975						0.47/0.47			0.89
A549	0.82	0.61	0.73		0.58	0.71/0.53	0.896	0.7	0.83
HCC827						0.35/0.55		0.67	0.74
H358	0.64							0.62	0.67
H2228			0.76						0.65
H3122			0.64						0.62
H460	0.64	0.84	0.54			0.53/0.72	0.518	0.76	0.61
H1650					0.4	0.26/0.23		0.27	0.57
CALU1				0.56					0.45
H520	0.49			0.48		0.49/0.83		0.54	0.49
H1437									0.45
H226	0.61	0.79		0.57					0.43
CALU3									0.32
H2009									0.23
H1781									0.18

Table S3. List of differentially expressed genes between radiosensitive and radioresistant cell lines.

GEN	Expression (log2FC)	GEN	Expression (log2FC)	GEN	Expression (log2FC)
AASS	-1.71	KCNS3	-1.66	TMEM144	1.99
ABCD3	1.15	KIAA1324L	-1.63	TNIK	-1.45
ADAMTS12	-1.30	KLHL24	-1.00	TRIM14	1.10
AHNAK2	2.29	LCA5	-1.02	TRIM6	1.80
ALDH6A1	-1.32	LINC00597	-1.20	TRIM7	1.67
APOBEC3B	2.76	LMO3	-1.25	USP18	1.05
ARG2	-1.66	LOC100287896	-1.27	USP43	1.24
ARHGAP42	1.01	LRIG1	-2.30	VWA5A	1.07

ATP6AP1L	-1.43	LRRC1	-1.01	ZC3H6	-1.06
BASP1	2.25	MAP9	2.07	ZNF114	2.56
BTNL9	1.41	MCM4	1.37	ZNF30	1.35
C4orf32	1.74	MLF1	-1.53	ZNF470	1.30
CD109	3.41	MPDU1	1.05	ZNF480	1.16
CDKN2B	-2.24	NANP	1.00	ABCC3	1.11
CDON	-1.00	NDRG4	1.27	ABCC5	-1.05
CELF2	-1.01	NGEF	1.36	DLL3	1.68
CEP70	-1.05	NTNG1	-1.42	MAN1A1	-2.05
CMTM3	1.43	PAG1	1.36	NUCB2	-1.09
CYBRD1	-1.51	PAIP2B	-1.39	PCDHB13	1.57
CYP27C1	1.33	PERP	1.06	PCDHB14	1.68
DVL3	-1.02	PFKFB4	1.17	PCDHB2	2.09
EDNRA	2.23	PHF19	1.85	PCDHB8	1.14
EGLN3	1.51	PPFIA3	1.06	CST3	1.64
ELOVL6	1.02	PSMB10	1.05	SPOCK1	3.11
EOGT	1.02	PXYLP1	-1.25	TNFAIP8	1.27
EPHX2	1.37	RFC3	1.01	TGFBI	2.24
FAM117A	-1.55	RHOBTB3	1.65	IGFBP5	-3.04
FARP1	-1.17	RMDN2	1.18	VEGFA	-1.05
FNBP1	1.06	RWDD2A	-1.34	FABP5	1.98
FYN	-2.24	SDR16C5	1.84	MCCC1	-1.34
GABRB3	2.06	SELENOP	-2.17	EMB	1.44
GALC	1.93	SEMA3A	2.05	CDH2	-2.43
GIMAP2	1.09	SLAMF7	1.30	MAGI2	-1.46
GOLM1	1.10	SLFN13	1.75	PPARA	-1.01
GPR160	-1.86	SNORA12	1.29	SH3RF2	1.57
GPRC5B	-1.55	SNX5	1.06	SYTL2	1.85
HECW2	1.50	SPEG	-1.32	NR3C2	-1.30
HENMT1	1.36	ST20	-1.11	PAQR8	-1.11
HOXB3	-1.37	ST3GAL3	-1.30	SPTBN1	1.12
HPGD	3.65	SYNPO2	1.07	TUBB3	1.25
JUN	-1.73	TM4SF18	2.41	JPH1	-2.19
KCNQ1OT1	-1.04	TMEM133	1.02	SYNM	1.81
				BMP5	1.27

Table S4. Summary of the characteristics of the 107 patients in the main study cohort.

Variables		N	%
Age at diagnosis			
	< 40 Years	1	0.9
	[40-49]	8	7.5
	[50-59]	28	26.2
	[60-69]	37	34.6
	≥70	32	29.9
	nd	1	0.9
Year of dianosis			
	<2000	5	4.7
	[2000-2009]	51	47.7
	≥ 2010	36	33.6
	nd	15	14
gender			
	woman	48	44.9
	male	59	51.4
Tumoral stage (AJCC)			
	Estadio I	24	22.4

	Estadio II	33	30.8
	Estadio III	50	46.7
Histology			
	Adenocarcinoma	55	51.4
	SCC	52	48.6
Status			
	alive	50	46.7
	death	57	53.3

Table S5. Results of the univariate analysis (log rank test) for potential prognostic and predictive factors of response.

Variable	Categories	n	Median OS (months)	p (Log Rank)	Median PFS (months)	p (Log Rank)
Age	< 63 years	47	37.6	0.346	25.38	0.457
	≥ 63 years	59	32		22.68	
	nd	1	-		-	
Year of diagnosis	< 2008	39	28.9	0,128	22.91	0.831
	≥ 2008	53	44.19		26.14	
	nd	15	-		-	
Sex	Male	59	37.58	0,42	26	0.612
	Female	48	32.45		22.75	
Histologic subtype	Adenocarcinoma	55	29.46	0.158	17.46	0.028
	SCC	52	43.89		29.98	
T component	T1 + T2	78	32.84	0.188	26	0.1
	T3 + T4	29	24.13		12.66	
Nodal load	n0-1	65	37.58	0.37	22.92	0.814
	n2-n3	40	27.45		17.62	
	nd	2	----		-	
Stage	I and II	57	43.89	0.051	26.96	0.183
	III	50	26.53		15.88	
Prognostic signature	Low risk	53	71.473	<0,001	47.11	<0.001
	High risk	54	24.131		12.65	
**Survival data cohort**						
Median OS (months): 32.7 (CI at 95% 25-40.4)						
Median PFS (months): 22.9 (CI at 95% 15-30.8)						
Median cancer-specific survival (months): 41.6 (CI at 95% 30.1-53.1)						

OS: overall survival; PFS: progression-free survival; CI= confidence interval; HR= hazard ratio; nd: not determined; N: sample number.

Table S6. Genes with a significant impact (p < 0.05) on overall survival measured by univariate Cox regression. They are shown in descending order by p value.

Gen	Beta	HR (IHR (95% CI)	wald.Wald test	Valor p value
APOBEC3B	-0.303	0.738 (0.623-0.875)	12.30	0.000456
GOLM1	0.352	1.42 (1.15-1.76)	10.20	0.001430
FAM117A	-0.473	0.623 (0.461-0.843)	9.43	0.002130
CDH2	0.283	1.33 (1.09-1.61)	8.26	0.004060
KCNQ1OT1	1.19	3.27 (1.44-7.43)	8.03	0.004600
SDR16C5	0.197	1.22 (1.05-1.41)	7.10	0.007730
KLHL24	-0.373	0.688 (0.512-0.926)	6.10	0.013500
PCDHB13	0.315	1.37 (1.06-1.78)	5.67	0.017300
PCDHB8	0.244	1.28 (1.04-1.56)	5.66	0.017400
PCDHB2	0.222	1.25 (1.04-1.5)	5.61	0.017900

RHOBTB3	0.24	1.27 (1.04-1.55)	5.49	0.019100
NUCB2	0.468	1.6 (1.08-2.37)	5.44	0.019700
BASP1	0.194	1.21 (1.03-1.43)	5.27	0.021800
TRIM6	0.284	1.33 (1.02-1.72)	4.58	0.032400
ABCC5	-0.166	0.847 (0.726-0.987)	4.52	0.033600
EPHX2	-0.272	0.762 (0.587-0.989)	4.18	0.040900
LRIG1	-0.283	0.754 (0.574-0.989)	4.16	0.041400
TMEM144	0.341	1.41 (1.01-1.95)	4.11	0.042500
PXYLP1	-0.373	0.688 (0.479-0.989)	4.08	0.043300
TUBB3	0.194	1.21 (1.01-1.47)	4.05	0.044100
USP43	0.252	1.29 (1-1.65)	3.92	0.047600

Beta= regression coefficient; HR= hazard ratio; CI= confidence interval;.

Table S7. Genes with a significant impact ($p < 0.05$) on PFS measured by univariate Cox regression. Genes are sorted in descending order by p value.

**Gene**	**Beta**	**HR (95% CI)**	**Wald test**	**P value**
GOLM1	0.467	1.6 (1.3-1.96)	20.20	7.00e-06
APOBEC3B	-0.323	0.724 (0.621-0.844)	17.00	3.75e-05
KCNQ1OT1	1.41	4.08 (1.82-9.15)	11.60	6.58e-04
PCDHB2	0.239	1.27 (1.08-1.5)	8.29	4.00e-03
TUBB3	0.257	1.29 (1.08-1.54)	8.13	4.37e-03
USP43	0.333	1.4 (1.11-1.76)	7.99	4.72e-03
NTNG1	0.369	1.45 (1.12-1.87)	7.78	5.29e-03
PCDHB8	0.259	1.3 (1.08-1.56)	7.76	5.35e-03
BASP1	0.209	1.23 (1.06-1.43)	7.55	6.01e-03
FARP1	0.429	1.54 (1.12-2.1)	7.29	6.95e-03
MPDU1	0.636	1.89 (1.19-3.01)	7.19	7.31e-03
ATP6AP1L	0.526	1.69 (1.14-2.51)	6.87	8.74e-03
PXYLP1	-0.413	0.662 (0.473-0.926)	5.80	1.60e-02
CYP27C1	0.229	1.26 (1.04-1.52)	5.77	1.63e-02
ABCC5	-0.17	0.844 (0.733-0.971)	5.59	1.81e-02
PAIP2B	0.319	1.38 (1.05-1.79)	5.54	1.86e-02
PCDHB13	0.279	1.32 (1.05-1.67)	5.52	1.88e-02
KLHL24	-0.33	0.719 (0.545-0.947)	5.51	1.90e-02
SDR16C5	0.159	1.17 (1.03-1.34)	5.47	1.94e-02
CDH2	0.208	1.23 (1.03-1.47)	5.44	1.97e-02
RHOBTB3	0.205	1.23 (1.03-1.47)	5.04	2.47e-02
HECW2	0.312	1.37 (1.04-1.8)	4.88	2.72e-02
FAM117A	-0.328	0.721 (0.538-0.966)	4.81	2.83e-02
SPTBN1	0.36	1.43 (1.03-1.99)	4.68	3.06e-02
CELF2	0.189	1.21 (1.01-1.45)	4.28	3.85e-02
JPH1	-0.212	0.809 (0.66-0.992)	4.13	4.21e-02
SLFN13	0.205	1.23 (1.01-1.5)	4.11	4.27e-02
ELOVL6	-0.212	0.809 (0.655-0.998)	3.93	4.75e-02
MLF1	-0.187	0.829 (0.688-0.998)	3.90	4.82e-02

Beta= regression coefficient; HR= hazard ratio; CI= confidence interval; ADC= adenocarcinoma; SCC= squamous cell carcinoma.

Table S8. Results of the analysis of the common gene signature by univariate Cox regression for OS and PFS.

**Histology**		**Beta**	**HR (95% CI for HR)**	**Wald test**	**P value**	**CI**
Lung NSCLC	(OS)	1.17	3.23 (2.11-4.95)	20.2	6.66e-08	0.71±0.0373

	(SLP)	1.24	3.46 (2.3-5.,2)	35.4	2.62e-09	0.72±0.032
ADC	(OS)	1.26	3.52 (1.85-6.69)	14.8	0.00012	0.69 ±0.055
	(SLP)	1.32	3.76 (2.03-6.97)	17.6	2.68e-05	0.72±0.047
SCC	(OS)	1.35	3.87 (1.91-7.83)	14.2	0.00016	0.73±0.05
	(SLP)	1.33	3.78 (1.93-7.42)	15	0.00011	0.73±0.048

Beta= regression coefficient; HR= hazard ratio; CI= confidence interval; ADC= adenocarcinoma; SCC= squamous cell carcinoma.

Table S9. Bivariate Cox regression analysis. Assessment of the effect of independent categorical variables on the HR of the prognostic gene signature of OS.

Variables	Beta	ET	Wald	HR	95,0% CI for HR		P value
					Low	High	
Signature for OS	1.09	0.28	15.04	2.99	1.72	5.20	0.00
Age at diagnosis	-0.34	0.27	1.58	0.71	0.42	1.21	0.21
Signature for OS	1.29	0.31	16.93	3.65	1.97	6.76	0.00
Year of diagnosis	0.52	0.31	2.88	1.69	0.92	3.09	0.09
Signature for OS	1.11	0.29	14.58	3.04	1.72	5.39	0.00
Gender	0.06	0.28	0.05	1.07	0.62	1.83	0.82
Signature for OS	1.20	0.32	13.69	3.32	1.76	6.26	0.00
Histologic subtype	-0.20	0.31	0.42	0.82	0.45	1.50	0.52
Signature for OS	1.11	0.28	15.50	3.03	1.74	5.26	0.00
T component	-0.42	0.29	2.10	0.65	0.37	1.16	0.15
Signature for OS	1.09	0.29	13.99	2.98	1.68	5.28	0.00
Nodal load	-0.02	0.28	0.01	0.98	0.57	1.68	0.93
Signature for OS	1.03	0.29	12.95	2.81	1.60	4.93	0.00
Tumor stage (AJCC)	-0.32	0.27	1.36	0.73	0.43	1.24	0.24

CI= confidence interval; HR= Hazard ratio

Table S10. Univariate Cox regression analysis. Impact of the independent categorical variables on the PFS.

Variables	Beta	ET	Wald	HR	95% CI for HR		P value
					Low	High	
Age at diagnosis	-0.18	0.25	0.55	0.83	0.51	1.35	0.46
Year of diagnosis	0.06	0.27	0.05	1.06	0.62	1.81	0.83
Gender	-0.12	0.24	0.25	0.88	0.55	1.43	0.62
Hystologic subtype	0.55	0.25	4.74	1.74	1.06	2.86	0.03
T Component	-0.44	0.27	2.63	0.64	0.38	1.10	0.10
Nodal Load	0.06	0.25	0.06	1.06	0.65	1.74	0.81
Tumoral stage (AJCC)	-0.32	0.24	1.76	0.72	0.45	1.17	0.18
Genetic signature SLP	1.30	0.27	23.45	3.66	2.16	6.18	<0.0001

CI= confidence interval; HR= Hazard ratio

Table S11. Bivariate Cox regression analysis. Assessment of the effect of independent categorical variables on the HR of the prognostic gene signature of PFS.

Variables	Beta	ET	Wald	HR	95% CI for HR		P value
					Low	High	
Signature for PFS	1.28	0.27	23.07	3.60	2.14	6.08	0.00
Age at diagnosis	-0.27	0.25	1.21	0.76	0.47	1.24	0.27
Signature for PFS	1.46	0.30	24.14	4.33	2.41	7.76	0.00
Year of diagnosis	0.09	0.28	0.12	1.10	0.64	1.89	0.73
Signature for PFS	1.31	0.27	23.50	3.72	2.19	6.33	0.00
Gender	0.10	0.25	0.16	1.11	0.68	1.81	0.69
Signature for PFS	1.33	0.30	19.13	3.79	2.09	6.89	0.00
Histologic subtype	-0.07	0.29	0.06	0.93	0.53	1.64	0.80
Signature for PFS	1.31	0.27	24.19	3.69	2.19	6.21	0.00
T component	-0.51	0.28	3.37	0.60	0.35	1.04	0.07
Signature for PFS	1.32	0.27	22.90	3.73	2.17	6.39	0.00
Nodal load	0.32	0.26	1.50	1.37	0.83	2.28	0.22
Signature for PFS	1.28	0.27	21.99	3.59	2.10	6.11	0.00
Tumor stage (AJCC)	-0.10	0.25	0.15	0.91	0.56	1.48	0.70

CI= confidence interval; HR= Hazard ratio

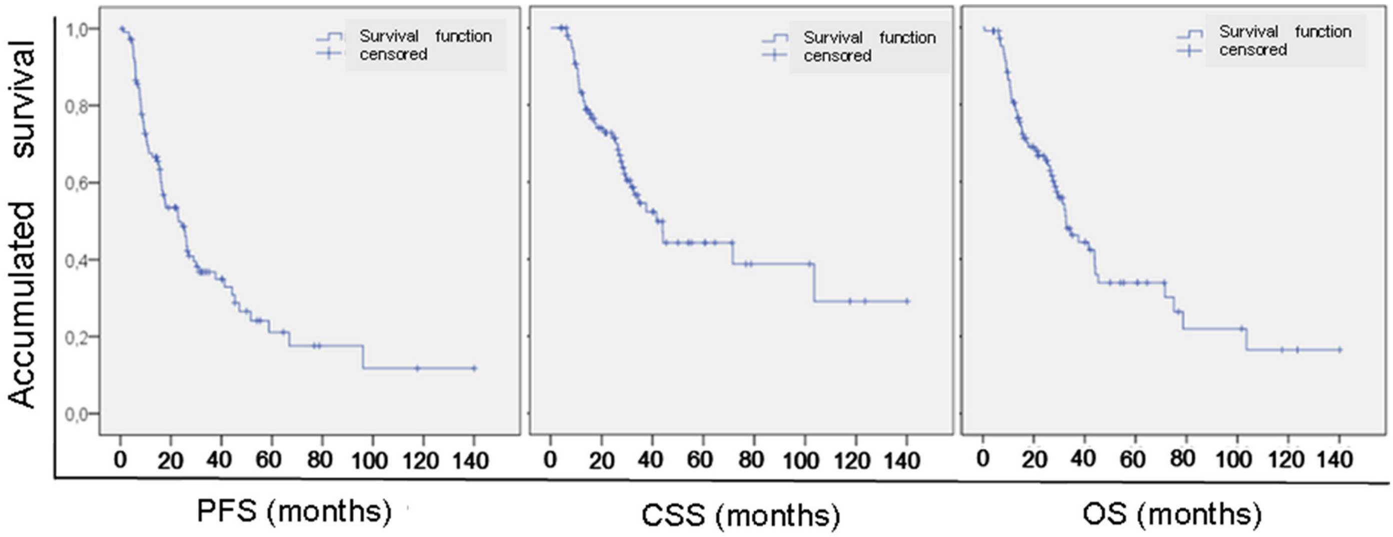

Figure S1. Kaplan Meier curves showing progression-free survival (PFS), cancer-specific survival (SCE), and overall survival (OS) of the cohort of interest.

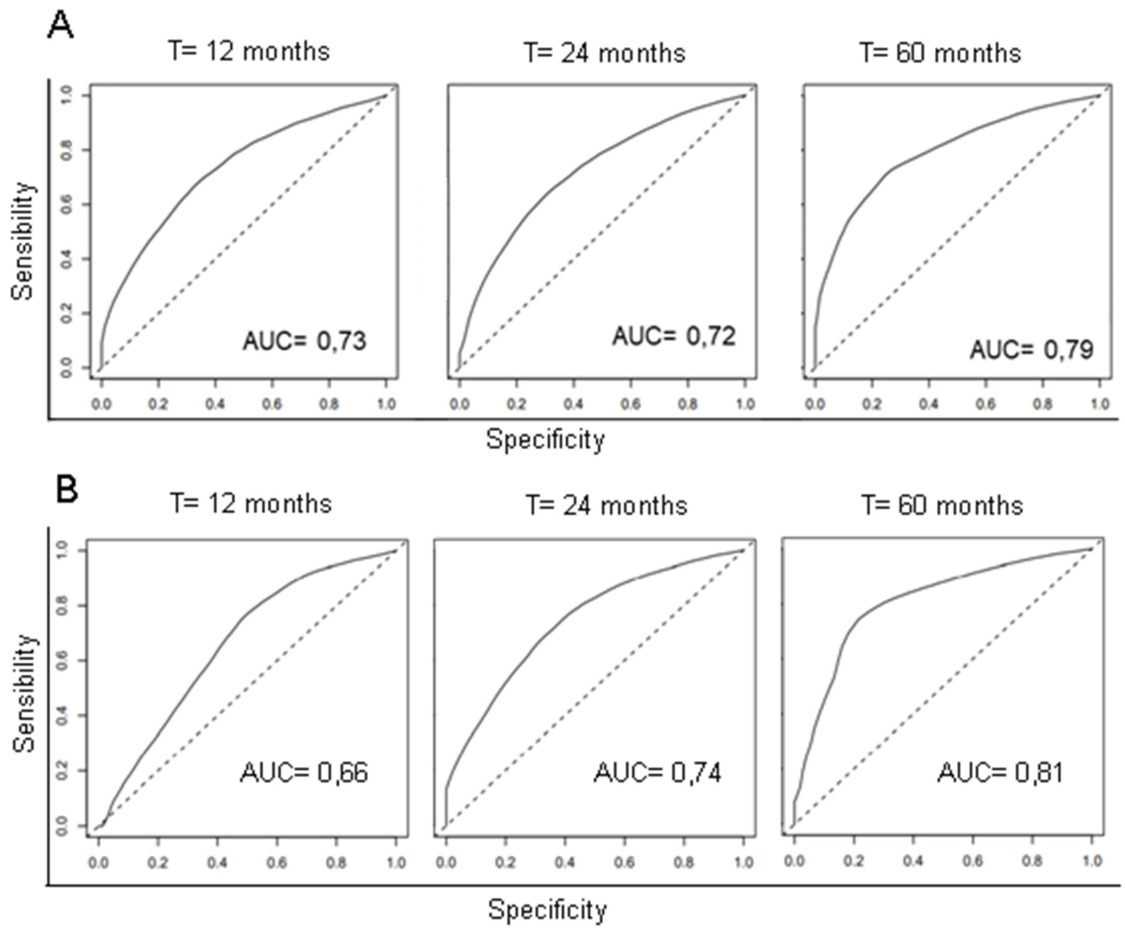

Figure S2. (A) Curvas ROC en función del tiempo (T). AUC: área bajo la curva. OS; (**B**) Curvas ROC en función del tiempo (T). AUC: área bajo la curva. PFS.

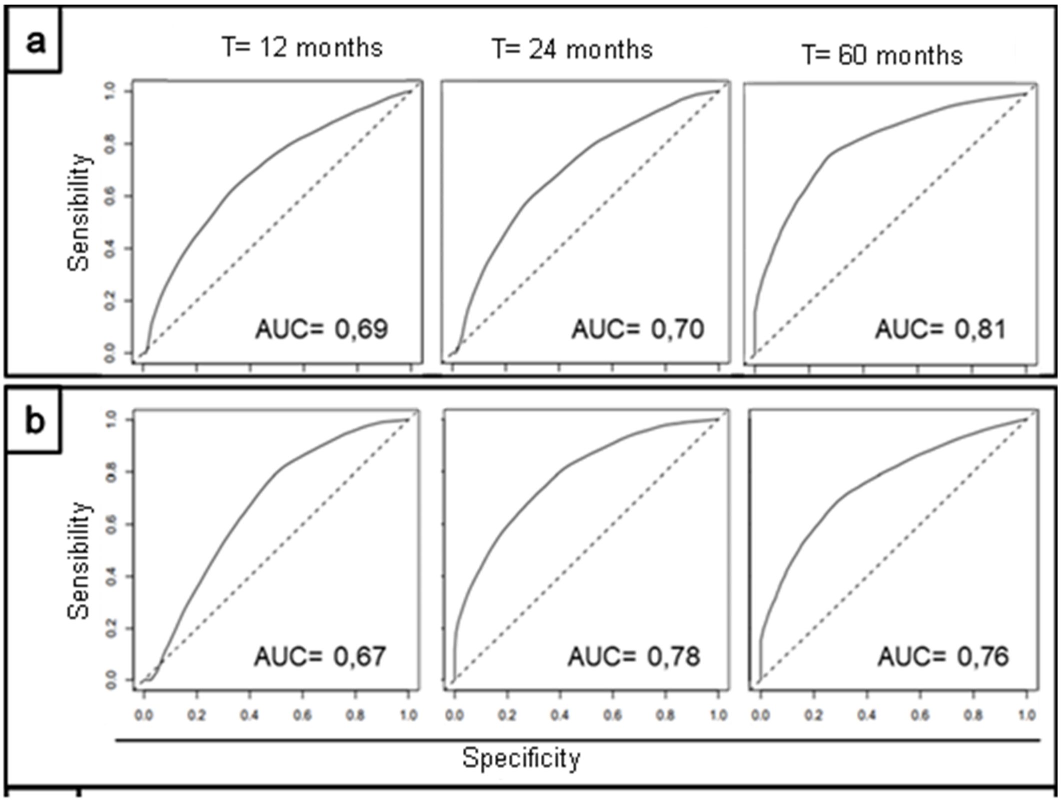

Figure S3. ROC curves at 12, 24 and 60 months of the common gene signature for SG (a) and SLP (b).

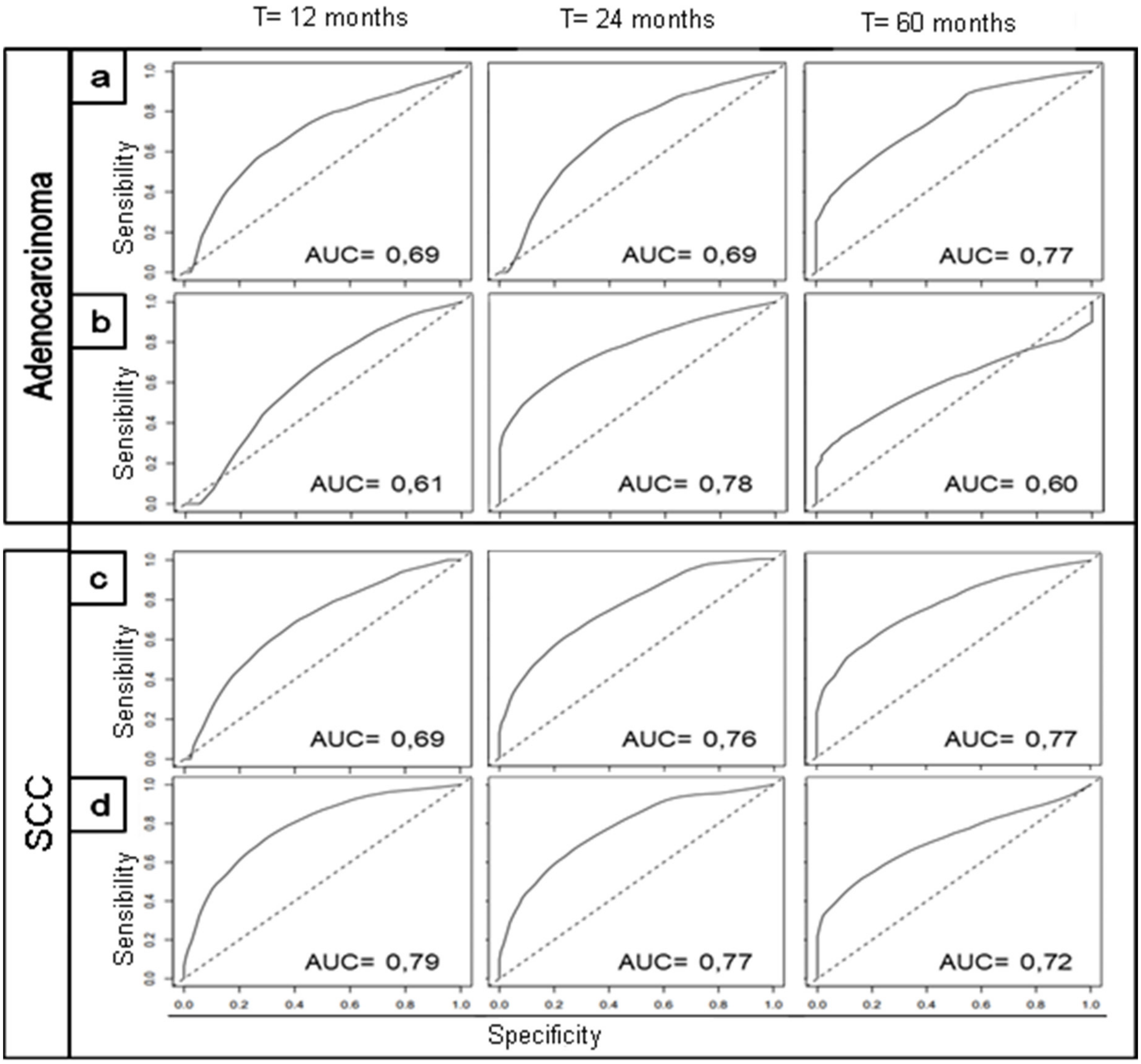

Figure S4. ROC curves at 12, 24 and 60 months of the common gene signature for SG (a and c) and SLP (b and d), according to the histologic groupAdenocarcinoma od squamous cell carcinoma (SCC).
